# Evaluation of Hybrid Models to Estimate Chlorophyll and Nitrogen Content of Maize Crops in the Framework of the Future CHIME Mission

**DOI:** 10.3390/rs14081792

**Published:** 2022-04-08

**Authors:** Gabriele Candiani, Giulia Tagliabue, Cinzia Panigada, Jochem Verrelst, Valentina Picchi, Juan Pablo Rivera Caicedo, Mirco Boschetti

**Affiliations:** 1 Institute for Electromagnetic Sensing of the Environment, National Research Council, 20133 Milan, Italy; 2 Remote Sensing of Environmental Dynamics Laboratory, University of Milano-Bicocca, 20126 Milan, Italy; 3 Image Processing Laboratory, University of València, 46980 València, Spain; 4 Research Centre for Engineering and Agro-Food Processing, Council for Agricultural Research and Economics, 20133 Milan, Italy; 5 Secretary of Research and Postgraduate, CONACYT-UAN, Tepic 63000, Nayarit, Mexico

**Keywords:** spaceborne imaging spectroscopy, radiative transfer modeling, machine learning regression algorithm, Gaussian process regression, active learning, chlorophyll content, nitrogen content

## Abstract

In the next few years, the new Copernicus Hyperspectral Imaging Mission (CHIME) is foreseen to be launched by the European Space Agency (ESA). This missions will provide an unprecedented amount of hyperspectral data, enabling new research possibilities within several fields of natural resources, including the “agriculture and food security” domain. In order to efficiently exploit this upcoming hyperspectral data stream, new processing methods and techniques need to be studied and implemented. In this work, the hybrid approach (HYB) and its variant, featuring sampling dimensionality reduction through active learning heuristics (HAL), were applied to CHIME-like data to evaluate the retrieval of crop traits, such as chlorophyll and nitrogen content at both leaf (LCC and LNC) and canopy level (CCC and CNC). The results showed that HYB was able to provide reliable estimations at canopy level (R^2^ = 0.79, RMSE = 0.38 g m^−2^ for CCC and R^2^ = 0.84, RMSE = 1.10 g m^−2^ for CNC) but failed at leaf level. The HAL approach improved retrieval accuracy at canopy level (best metric: R^2^ = 0.88 and RMSE = 0.21 g m^−2^ for CCC; R^2^ = 0.93 and RMSE = 0.71 g m^−2^ for CNC), providing good results also at leaf level (best metrics: R^2^ = 0.72 and RMSE = 3.31 μg cm^−2^ for LCC; R^2^ = 0.56 and RMSE = 0.02 mg cm^−2^ for LNC). The promising results obtained through the hybrid approach support the feasibility of an operational retrieval of chlorophyll and nitrogen content, e.g., in the framework of the future CHIME mission. However, further efforts are required to investigate the approach across different years, sites and crop types in order to improve its transferability to other contexts.

## 1 Introduction

The Hyperspectral Precursor of the Application Mission (PRISMA) satellite [1,2], successfully launched in 2019 by the Italian Space Agency (ASI), opened a new era of imaging spectroscopy from space. In the next few years, several other hyperspectral missions will join PRISMA. The list includes the Hyperspectral Imager Suite (HISUI) [3] from the Japanese ministry of Economy, Trade, and Industry (METI), operational from 2021, the Environmental Mapping and Analysis Program (EnMap) [4] from the German Space Agency (GFZ-DLR), planned to be launched in 2022, the Spaceborne Hyperspectral Applicative Land and Ocean Mission (SHALOM) [5] from the Italy-Israel Space agencies (ASI-ISA), expected to be launched in 2022, the HypXIM satellite [6] from the French Space Agency (CNES), the Surface Biology and Geology (SBG) mission [7] from the National Aeronautics and Space Administration (NASA) and the Copernicus Hyperspectral Imaging Mission (CHIME) [8] from the European Space Agency (ESA), scheduled for the late-2020s.

All these spaceborne imaging spectrometers will acquire radiometric data within the visible-to-shortwave infrared spectral domain (between 400 nm and 2500 nm, approximately) and with a spatial resolution ranging from 20 m to 30 m. These missions will provide an unprecedented amount of spaceborne hyperspectral data, enabling new research possibilities within several fields of natural resources studies, including atmosphere, ocean and land applications. In particular, CHIME will provide hyperspectral observations with high radiometric accuracy in the range of 400–2500 nm, at a spatial resolution of 20–30 m and with a revisit time of 10–12.5 days, using a sun synchronous orbit with an overpass time between 10:30–11:30 LTDN (Local Time on Descending Node) [9]. The main objective of the CHIME mission is to provide routine hyperspectral observations through the Copernicus Program, with the purpose to develop new and improved Copernicus services with a focus on the precise management of natural resources to support the monitoring, implementation and improvement of a range of related policies and decisions [9]. Within the natural resources pillar, “agriculture and food security” is the primary application domain selected to meet user requirements in agricultural services and sustainable agricultural management and to support EU-related policies, such as the EU Common Agricultural Policy [10]. Regarding this aspect, the choice of 20–30 m as CHIME spatial resolution was based on a previous user requirement study founded by ESA [9]. From the results of this study, the CHIME Mission Advisory Group considered 20–30 m a good compromise between technical constraints and user needs and a suitable spatial resolution to provide useful information related to the “agriculture and food security” domain. In order to efficiently exploit this upcoming hyperspectral data stream, new processing methods and techniques need to be studied and implemented.

For the sustainable use of nutrients, the CHIME mission requirement document selects chlorophyll content (C_ab_) as an essential eco-physiological variable for photosynthetic functioning and a major parameter for the monitoring of plant nitrogen uptake (PNU) during crop development. Temporal and spatial estimation of PNU is an essential information to assess crop nutritional status, in order to support a more efficient fertilization by supplying the plants with the requested amount of nitrogen (N): this will sustain high-quality yields as well as minimize nitrate leaching to the groundwater [9]. Many studies in the literature estimated PNU exploiting the relation between N and C_ab_, measured from either remotely sensed C_ab_ [11,12] or measured in field by chlorophyll meter sensors [13–16]. In a previous review paper, Homolova et al. [17] showed that the existing C_ab_-N relation is moderately strong across different species (average Pearson correlation coefficient equal to 0.65 ± 0.15), highlighting that this positive relation is species specific across different types of plants. In a recent review paper, Berger et al. [18] discussed some limitations of commonly used C_ab_-N relation, suggesting the exploration of potentially more robust alternatives for PNU monitoring. Taking into account that (i) the largest amount of leaf N is contained in proteins and (ii) a non-linear relation exits between C_ab_ and N throughout the growing season, the authors proposed the use of protein content (Cp) as a direct way to estimate N in plants, using 4.43 as the nitrogen-to-protein conversion factor [19].

Traditionally, the estimation of C_ab_ and N (and vegetation traits in general) from remotely sensed data was mainly performed through parametric and non-parametric regression methods. In the first approach, an arithmetic combination of reflectance bands (e.g., a vegetation index) is usually linked to the trait of interest through a fitting model. In the second approach, the fitting model between reflectance bands and the trait is non-explicit and it is directly defined by the method itself (e.g., machine learning (ML) regression algorithms). See Verrelst et al. [20,21] for a detailed overview of these methods as well as Berger et al. [18] for N retrieval methods, specifically. Despite the good results and the computational efficiency provided by these methods, the identified fitting models are typically valid only for their respective case studies, lacking transferability to other contexts.

Conversely, physically based methods represent an interesting alternative to provide robustness and transferability [18]. These methods exploit radiative transfer models (RTMs), mathematical equations based on physical laws which describe light absorption and multiple scatterings in order to simulate vegetation reflectance spectra, starting from leaf biophysical variables and canopy structure. In order to estimate crop traits (e.g., N), RTM-simulated spectra need to be inverted. However, this inversion can be challenging due to a number of unknowns larger than the number of independent observations, which makes the inversion an undetermined problem. To overcome these issues, the RTMs inversion can be performed through either iterative numerical optimization methods or inversion based on lookup tables (LUTs) [21]. Although these approaches have been already successfully exploited [22–26], both methods remain computationally expensive.

In the last few years, the scientific community proposed the hybrid approach as a possible solution to the drawbacks of regression algorithms and physically based methods [27–30]. This approach includes elements of both non-parametric and physically based methods: RTMs are exploited to simulate an LUT of thousands of vegetation reflectance spectra; the generated LUT together with the corresponding crop traits represent the input–output pairs used to train the ML regression algorithms. Therefore, the hybrid approach features the generic properties of physically based methods as well as the flexibility and computational efficiency of the non-parametric non-linear ML methods.

When dealing with hyperspectral datasets to train a model, the high dimensionality can lead to both collinearity and long computational time issues, even in the case of a hybrid approach. In the spectral domain, feature reduction methods (e.g., principal component analysis) can be used to reduce noisy and redundant bands. In the sampling domain, intelligent sampling schemes based on the Active Learning (AL) technique, as proposed in Verrelst et al. [31,32], can be used to optimize the spectra cardinality of the LUT. The heuristics used in AL select only the most informative samples based on either diversity or uncertainty metrics [33], avoiding possible unrealistic conditions due to the random selction of RTM input parameters. AL techniques within the hybrid framework were recently applied to PRISMA data, providing excellent results for the mapping of canopy nitrogen content [34] and other crop traits [35] on rural regions.

The goal of this study, performed within the framework of the CHIME Mission Requirements Consolidation Study (CHIME-RCS), is the evaluation of the hybrid approach for the retrieval of C_ab_ and N content from hyperspectral data, comparing several combinations of spectral and sample dimensionality reductions. The hybrid approach was selected due to its potential for operational products generation. C_ab_ and N were selected as important parameters for the user community due to their wide use in agricultural monitoring studies devoted to assess actual crop nutritional status in precision farming applications. C_ab_ and N content was estimated applying the hybrid method to a synthetic CHIME datasets, simulated from two images acquired by the airborne hyperspectral sensor Hyplant-DUAL, during the 2018 ESA FLEXSENSE campaign held in Grosseto, Italy.

## 2 Materials and Methods

### 2.1 Study Area and Field Campaigns

**Figure 1:**
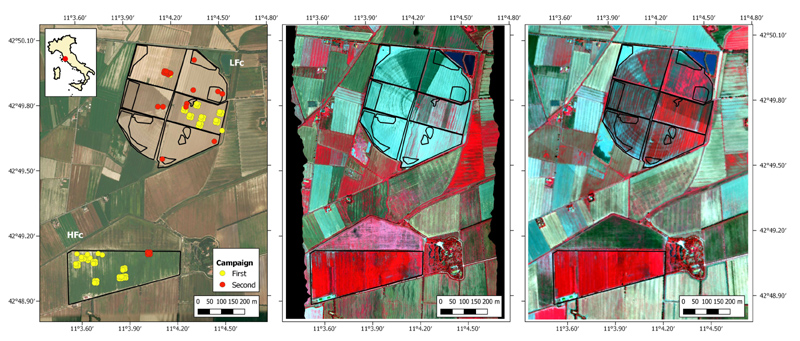
Study area (left) and EO data acquired by HyPlant-DUAL on 7 July (center) and 30 July (right) 2018. The yellow and red dots overlaid on the study area represent Elementary Sampling Units (ESU) measured during the two field campaigns.

The study area (Figure 1) is located in Tuscany (Central Italy), North of Grosseto (42°49′47.02″N 11°04′10.27″E; elev. 2 m a.s.l.). The site consists of a large flat area with an annual average temperature around 15 °C and annual average rainfall around 640 mm. Within the study area, two maize fields covering a total extension of more than 100 ha, from two different farms (Le Rogaie and Ceccarelli), were selected as test sites. At the time of aerial and ground surveys, these fields featured different conditions in terms of crop traits, due to different irrigation systems (Pivot system for Le Rogaie and micro drip for Ceccarelli) and different sowing dates. In particular, due to its large extension, Le Rogaie field was divided into six sectors, each sown at different dates. Ceccarelli fields were planted in early May whereas Le Rogaie fields were sown from mid to end of June, after the harvest of winter ryegrass. For this reason, in the following sections, the two fields are referred to as low and high vegetation fractional cover fields (LFC and HFC for Le Rogaie and Ceccarelli, respectively).

Two field campaigns (from 2 to 7 July and from 31 July to 1 August 2018) were carried out in order to measure crop traits, such as Leaf Area Index (LAI), leaf chlorophyll content (LCC) and leaf nitrogen content (LNC). The sampling strategy was performed following international protocols and guidelines, as proposed by the CEOS LPV group [36] and the VALERI project [37]. Crop trait measurements and leaf samples were acquired within 87 Elementary Sampling Units (ESU), each one covering an area of 10 × 10 m^2^.

Leaf biochemical variables were measured by sampling the last fully developed leaf from 3 plants with a subset of 31 ESUs, for a total of 93 independent samples at leaf level. Additional leaf samples in chlorosis condition (11), not corresponding to any specific ESU, were collected to broaden the range of variability, increasing the total number of analyzed leaves to 104. Table 1 provides, for each crop trait exploited in this study, a summary of acquired data, their units and measurement methods.

LCC laboratory extractions were performed on a set of three disks with 2.2 cm diameter (total area 11.40 cm^2^) sampled from each leaf. Each sample leaf material (3.8 cm^2^) was homogenized (Ultra-Turrax, IKA-Werk, Staufen, Germany) in 5 mL of ice-cold methanol for one minute. The homogenized samples were kept at −20 °C for twenty minutes. After centrifugation (4500 rpm for 10 min at 4 °C), the supernatant was recovered and kept at −20 °C. The pellet was re-extracted in 5 mL ice-cold methanol, kept for ten minutes at −20 °C and then centrifuged. This operation was repeated twice. Finally, the three supernatants were merged, and 2 mL were filtered (0.45 μm PTFE syringe filter) and immediately analyzed for chlorophyll content. All preparations and analyzes were carried out at low temperature and dim light. Moreover, for all the 87 ESUs, indirect measurements of leaf chlorophyll were acquired using a SPAD-502 chlorophyll meter (Konica Minolta, Tokyo, Japan). LCC values from laboratory extractions and the corresponding SPAD measurements were used to identify the SPAD-LCC relation (*R*^2^ = 0.93):

(1) $$\text{LCC}=8.24_{e}{}^{\left(0.0324.SPAD\right)}[\mu g\;cm^{-2}]$$

The relation in Equation (1) was then applied to SPAD data to estimate LCC on all the 87 ESUs.

A second set of disks, sampled from the same leaves, was used for the estimation of leaf nitrogen concentration (N_mass_ [%]), leaf mass per area (LMA [g cm^−2^]) and other crop traits not used in this study (e.g., equivalent water content [cm]). The leaf disks were oven-dried at 80 °C for 24 h, then weighted using an analytical scale (sensitivity: 0.0001 g) to measure the dry biomass. Vegetation material was then prepared for nitrogen estimation, using an electric mini chopper to homogenize the sample. N_mass_ was determined by dry combustion with a CN elemental analyzer (Flash EA 1112 NC-Soil, Thermo Fisher Scientific, Pittsburg, PA, USA). Leaf Nitrogen Content (LNC) was calculated from N_mass_ and LMA according to the following equation:

(2) $$\text{LNC}=10.{\text{N}}_{mass}.\text{LMA}[\text{mg}\;{\text{cm}}^{-2}]$$

In order to derive Canopy Chlorophyll Content (CCC) and Canopy Nitrogen Content (CNC), LAI was measured in 87 ESUs using either LAI2200 plant analyzer (LI-COR Inc., Lincoln, NE, USA) or digital hemispherical photography [38,39], according to plant development stage. LAI2200 data were acquired along 10 m transects following the ABBBBA sequence of above (A) and below (B) canopy measurements. LAI data were then post-processed using the software FV2200. Hemispherical photography was performed using a CoolPix 990 4 Megapixel digital camera (Nikon, Tokyo, Japan ), equipped with a FE-E8 8 mm fish-eye lens (Nikon, Tokyo, Japan) and mounted on a tripod. The pictures were acquired at the center and at the four corners of each ESU. During the first campaign, downward pictures were taken in ESUs with small maize plants (development stage V2–4), whereas upward modality was used in the second campaign. The hemispherical pictures were post-processed through Can-Eye v6.4.91 [39] to estimate LAI values which were averaged for each ESU.

Finally, CCC and CNC were calculated multiplying the corresponding LCC and LNC by LAI:

(3) $$\text{CCC=}\frac{1}{100}\cdot \text{LCC}\cdot \text{LAI}\;{\text{[gm}}^{-\text{2}}\text{]}$$

(4) $$\text{CCC=}10\cdot \text{LCC}\cdot \text{LAI}\;{\text{[gm}}^{-\text{2}}\text{]}$$

### 2.2 Earth Observation Dataset

The Earth Observation (EO) dataset is represented by two hyperspectral images acquired by the HyPlant-DUAL instrument [40–42] on 7 and 30 July. HyPlant is a novel airborne imaging spectrometer, developed by the Jülich Forschungszentrum in cooperation with SPECIM Spectral Imaging Ltd (Oulu, Finland). The spectrometer consists of two hyperspectral modules able to measure reflectance (DUAL) and sun-induced chlorophyll fluorescence, operating in a push-broom modality. The DUAL imager includes two sensors, integrated in a single housing and with the same fore optics, providing contiguous spectral information from 370 to 2500 nm with 3–10 nm spectral resolution in the VIS/NIR spectral range and 10 nm spectral resolution in the SWIR spectral range. The technical features of the acquired images are summarized in Table 2. The study area was covered by six different flight lines on 7 July and by four flight lines on 30 July, with a ground sampling distance (GSD) of 1 m and 4.5 m, respectively. HyPlant-DUAL images were provided georectified and atmospherically corrected to top-of-canopy reflectance [43]. In order to evaluate the potential of CHIME to assess crop traits, these images were spectrally resampled to CHIME-like bands, according to theoretical Gaussian spectral response functions (i.e., 210 bands with 10 nm bandwith). The bands influenced by atmospheric water vapor absorption were removed, leading to a final CHIME-like spectral configuration of 157 bands. CHIME-like reflectance spectra, used in the validation step of retrieval models, were collected from the images with the original GSD (1 m and 4.5 m), at the same locations of field measurements. Every ESU location was visually checked and repositioned, if necessary, to match the right location of field measurements. For each ESU, the relative reflectance was calculated as the average reflectance of all pixels falling within the ESU extent (10 m). Moreover, for mapping demonstration, the images were spatially resampled to the expected CHIME spatial resolution of 30 m, through a cubic convolution algorithm, to provide realistic CHIME maps of estimated crop traits.

### 2.3 Crop Trait Retrieval

Crop trait retrieval was performed following a hybrid approach (HYB). According to this scheme, a radiative transfer model is used to simulate canopy reflectance, considering leaf and canopy vegetation parameters as well as background, illumination and viewing conditions (Sun–target–sensor geometry). Then, the RTM is run in forward mode to generate a database (LUT), which includes input-output pairs corresponding to crop traits of interest (i.e., LCC, LNC, CCC and CNC) and the related simulations of reflectance spectra. The generated LUT is then analyzed using ML regression algorithms to define a predictive relation between reflectance spectra and crop traits. The ML regression algorithm used in this work is the Gaussian process regression (GPR), which has been successfully exploited in several previous studies [26,44–46]. Moreover, the hybrid approach optimized through the active learning technique (HAL), recently introduced for the retrieval of aboveground nitrogen content [31,32], was evaluated and compared to the results obtained by the standard HYB approach. For both HYB and HAL, also several spectral dimensionality reduction strategies were tested, using the Principal Component Analysis with different numbers of components (PCA5, PCA10, PCA15, PCA20). The following sections provide further details on the RTM used in this study, the GPR algorithm and the AL heuristics.

#### 2.3.1 PROSAIL-PRO Radiative Transfer Model

The RTM, used to test the performance of hybrid approaches to retrieve crop traits, is the latest release of PROSPECT, the PROSPECT-PRO model [47]. The novelty of this model is the introduction of nitrogen-based constituents (proteins–C_p_) and carbon-based compounds (CBC), which replace the traditional dry matter content (C_m_). Proteins are the base parameter used for the retrieval of nitrogen content at both leaf (LNC) and canopy (CNC) level. Since PROSPECT-PRO simulates reflectance and transmittance spectra at leaf level, a MATLAB script was specifically developed to couple PROSPECT-PRO with the 4SAIL canopy model [48,49]. The 4SAIL model describes the canopy as a homogeneous medium where leaves are randomly distributed, using a limited number of structural variables in addition to leaf reflectance and transmittance and background (BG) reflectance (e.g., soil). The resulting model (PROSAIL-PRO) is able to simulate reflectance spectra at canopy level.

**Figure 2:**
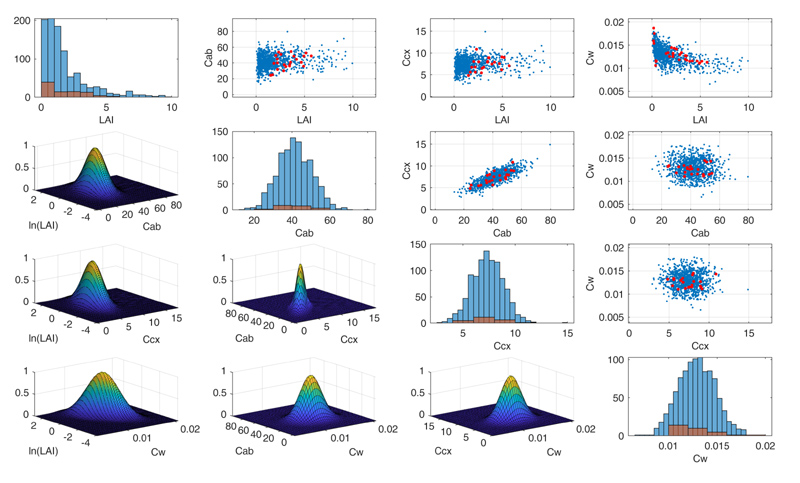
Example of PROSAIL-PRO random inputs generated from a multivariate normal PDF, considering covariance values among plant traits. Red dots in the scatter plots represent values measured during the Grosseto 2018 campaign; the blue dots are randomly sampled values used as input for the PROSAIL-PRO simulations.

The generation of the LUT used to train ML algorithms represents a critical step as it should be representative of vegetation reflectance spectra, including a priori information on the distribution of the input variables [50]. To avoid unrealistic combinations of PROSAIL-PRO input variables, a MATLAB script was designed to exploit covariances between some of the maize parameters measured during the Grosseto 2018 campaign (Figure 2). Moreover, for each crop parameter, the script allows the user to choose between several two-parameter families of Probabilities Density Functions (PDFs), such as Uniform distribution and Gaussian distributions. Most of the PDFs and the related ranges were selected according to actual values observed during the field campaign; the remaining values were selected according to literature or authors experience. The full PROSAIL-PRO parameterization is summarized in Table 3.

To test HYB and HAL approaches, random values were sampled from the selected PDFs and passed as input to the PROSAIL-PRO model. The generated LUT included 2000 reflectance spectra and the corresponding crop traits. This LUT size, which may seem small compared to other ML algorithms such as Neural Networks, was considered a good trade-off between representation of vegetation traits’ variability and computation time during the training phase. This is justified considering that for kernel-based algorithms—such as GPR—a relatively small training dataset is sufficient to identify the non-linear relations between spectral observations and the variables of interest [34]. This has been also confirmed by other studies using similar sampling sizes for GPR training datasets. In [46], the impact of the training database size was analyzed by building retrieval models based on LUT sizes of 1000, 2000, 3000, 5000 and 10,000 simulations. A training database of 3000 samples was chosen as a fair compromise between accuracy and processing time. A similar analysis was performed in [51], where the influence of the LUT size (ranging from 1000 to 10,000 with a step size of 1000 samples), on retrieval accuracy and training time, was investigated. In this case, an LUT of 2000 samples was considered a good trade-off between accuracy and time.

#### 2.3.2 Gaussian Process Regression

Gaussian process regression is a non-linear non-parametric regression algorithm which falls under the kernel-based function category. GPR, based on Gaussian processes (GPs), generalizes Gaussian probability distributions in a function space [52]. Differently from the majority of machine learning algorithms, the training phase in GPR is based on a Bayesian framework, leading to probabilistic outputs [21]. Introduced in Rasmussen [53], GPR was first applied in the field of hyperspectral data to map vegetation traits by Verrelst et al. [44,54]. According to Caicedo et al. [55], the recent successful exploitation of GPR in the vegetation traits mapping is related to two main features: (i) the algorithm is able to provide both a predictive mean and a predictive variance (uncertainty) and (ii) the algorithm is able to use very sophisticated kernel functions to model covariance.

Basically, GPR models the relation between input samples $x\in {\mathbb{R}}^{D}$ (i.e., CHIME-like spectra) and output observations x∈ℝ (i.e., a specific vegetation trait) as y=f(x)+ϵ, where *ϵ* is an additive Gaussian noise with zero mean and variance $\sigma_{n}^{2}$, and *f* (**x**) is a Gaussian-distributed random vector with zero-mean and covariance matrix **K**(**x**, **x**), i.e., f(x)∼N(0,k). The covariance matrix takes into account the main statistical properties of the variable to be modeled through a user-defined kernel function matrix *k*(**x**_*i*_, **x**_*j*_), encoding the similarity between each combination of the input samples **x_*i*_** and **x***j*. In this study, the asymmetric Square Exponential (SE) was used as a kernel function due to its capability to (1) approximate smoothly varying functions and (2) deal with asymmetries in the feature space [56]:

(5) $$k(x_{i},x_{j})=\sigma_{s}^{2}\exp(-\frac{1}{2}\sum_{b=1}^{D}{\left[\frac{x_{i}(b)-x_{j}(b)}{\sigma_{b}}\right]}^{2}),$$

where $\sigma_{s}^{2}>0$ represents the output variance, while *σ_b_* is related to the spread of the training information along the input dimension *b*. The covariance matrix is completely defined once $\theta =\{\sigma_{s}^{2},\sigma^{2},\sigma_{s}^{2}\}$ is set. The Bayesian framework of GPR allows estimating the distribution of *f* at any test point ***x***_∗_(i.e., a new pixel) conditioned on the information carried by the training data. According to its formulation, *f* (***x***_∗_) is normally distributed with a mean and variance given by:

(6) $$\begin{array}{l} f(x_{*})=k_{*}^{T}{\left(K+\sigma_{n}^{2}I_{N}\right)}^{-1}y\\ \sigma_{f}^{2}(x_{*})=c_{*}-k_{*}^{T}{\left(K+\sigma_{n}^{2}I_{N}\right)}^{-1}k_{*} \end{array}$$

where *N* is the number of available training samples, $y={\left[y_{1},...,y_{N}\right]}^{T}$ is the training output, $c_{*}=k(x_{*},x_{*})+\sigma_{n}^{2}$ and $k_{*}={\left[k(x_{*},x_{1}),\ldots ,k(x_{*},x_{N})\right]}^{T}$ is an *N* × 1 vector containing the similarity between ***x***_∗_and the training input information. The probability of the observations given the model hyperparameters p(y|x,θ) is provided by the marginal likelihood over the function values *f*, whose maximization during GPR training directly provides the optimum value of ***θ*** [57]. Finally, once optimized ***θ*** is known, the prediction of *y* for any input ***x***_∗_, along with its uncertainty, is given by Equation (6).

The GPR algorithm used in the training phase of this study is the MATLAB version implemented in the MLRA toolbox of the Automated Radiative Transfer Models Operator (ARTMO, https://artmotoolbox.com/, (accessed on 1 September 2021); [55,58]). Several options and kernel functions implemented in the Matlab version help optimizing and speeding up the training process, which becomes a relevant factor when GPR is implemented in an iterative process such as AL [34]. For this study, the GPR was set up using the default ARTMO settings, which include exact fit methods, constant basis functions and a qr computation method. In the case of LNC, the default parameters were not suited to identify a proper model; therefore, they were set through an optimization procedure implemented in ARTMO. In addition, the model was trained using a cross-validation method with 10 k-folds and 5% Gaussian noise was added to both parameters (crop traits) and simulated spectra, in order to generalize the retrieval model.

#### 2.3.3 Active Learning Approach

The active learning technique was applied within the hybrid framework (HAL) to evaluate its performance in the retrieval of selected maize traits and to compare its results to those obtained using the standard HYB approach. The AL technique is a dimensionality reduction (DR) approach which optimizes the number of samples in the LUT through an intelligent sampling strategy. In the first step, a small pool of reflectance spectra, randomly selected from the original LUT (e.g., 20 samples), is used to train a GPR model and to evaluate its performance against the validation data. Then, iteratively, a new sample is added to the reduced LUT—according to an AL heuristic—and a new GPR model is trained: if the new sample improves the model validation statistics, it is kept in the training pool, otherwise it is rejected. This procedure is performed for all the simulated reflectance spectra available in the original LUT. The final result is a reduced LUT including only the spectra that increased the model performance. The AL heuristics tested in this work belong to two different groups: criteria based on uncertainty, which include samples with greater disagreements between the different explanatory variables, and criteria based on diversity, which include samples distant from the selected training LUT. The first group includes variance-based pool of regressors (PAL) [59] and residual regression AL (RSAL) [60]; the second group includes Euclidean distance-based diversity (EBD) [61], angle-based diversity (ABD) [62], and cluster-based diversity (CBD) [63]. All the tests were performed using the AL module implemented in the ARTMO toolbox.

## 3 Results

Validation results, obtained from the comparison of estimated crop traits (for both HYB and HAL) with field measurements, are summarized in Table 4: accuracy and goodness-of-fit metrics are expressed in terms of coefficient of determination (R^2^), Root Mean Square Error (RMSE) and RMSE normalized respect to range (defined as the maximum value minus the minimum value) of measured data (nRMSE). In case of HAL, results are reported only for the best performing AL algorithm for each trait and spectral DR combination.

HYB showed good results at canopy level, where both CCC and CNC traits were assessed with high accuracy for all DR. The best performances were represented by R^2^ = 0.79, RMSE = 0.38 g m^−2^ and nRMSE = 13.40% for CCC with PCA05 and R^2^ = 0.84, RMSE = 1.10 g m^−2^ and nRMSE = 11.93% for CNC with PCA10. Conversely, very poor results were observed at leaf level: in this case, the GPR was not able to identify a suitable model to assess LCC and LNC traits.

The addition of active learning step into the hybrid approach represented a major improvement for crop trait retrieval, increasing goodness-of-fit metrics, especially at leaf level. As in the case of HYB, canopy level traits were assessed with better accuracy than the corresponding leaf level traits. The results at canopy level are comparable among all tested PCA configurations (best metrics: R^2^ = 0.88, RMSE = 0.21 g m^−2^ and nRMSE = 7.54% for CCC with PCA10; R^2^ = 0.93, RMSE = 0.71 g m^−2^ and nRMSE = 7.69% for CNC with PCA10). At leaf level, only some DR configurations provided good results. Regarding LCC, the best performance was achieved for 10 and 20 PCA components (best metrics: R^2^ = 0.72, RMSE = 3.31 μg cm^−2^ and nRMSE = 11.32%). In case of LNC, 10 and 15 components provided the best results (R^2^ = 0.55–0.56, RMSE = 0.02 mg cm^−2^ and nRMSE = 16.36–16.69%).

**Figure 3:**
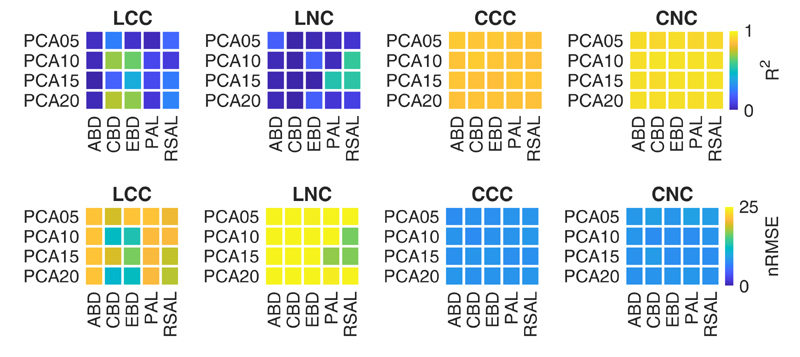
Metrics of crop traits estimation through HAL for different combinations of AL heuristics and spectral DR configurations, in terms of R^2^ and nRMSE.

Focusing the analysis on HAL, Figure 3 shows the impact of different DR combinations of AL heuristics and PCA configurations, in terms of R^2^ and nRMSE. The figure well depicts how crop traits are better retrieved at canopy level respect to the leaf level. More in details, the figure shows that HAL is able to provide very good results for all DR combinations at canopy level. Conversely, only few DR combinations provided good results at leaf level: for LCC, the best estimates were achieved using CBD and EBD with either 10 or 20 components; in case of LNC, only PAL with 15 components and RSAL with either 10 or 15 components provided satisfactory results.

**Figure 4:**
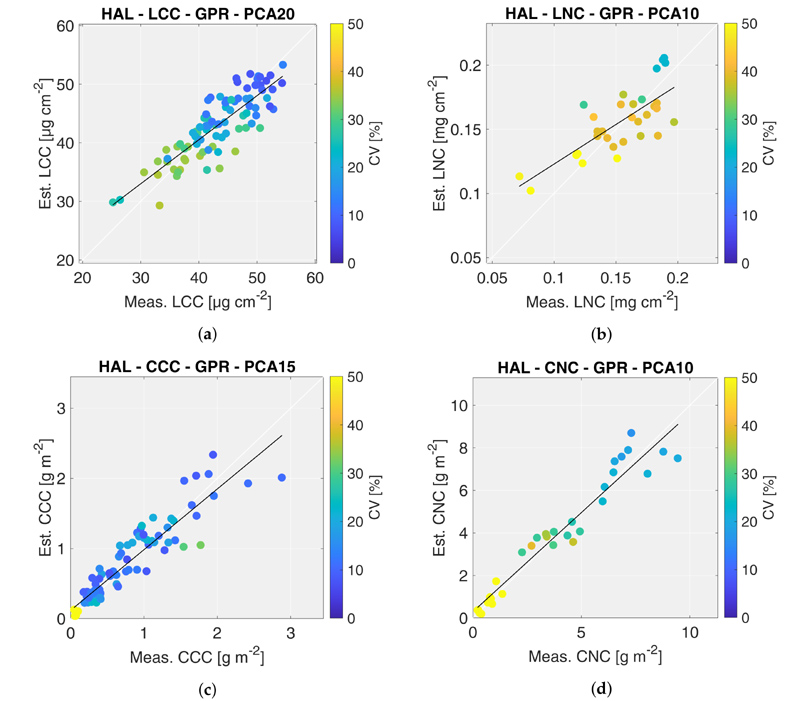
Scatter plots of the best HAL models: leaf chlorophyll content (a), leaf nitrogen content (b), canopy chlorophyll content (c), canopy nitrogen content (d). The colors of points represent the coefficient of variation (CV) estimated by the GPR algorithm.

Based on the results shown above, the scatter plots of the best HAL models are reported in Figure 4, where the colors of the points represent the coefficient of variation (CV) estimated by the GPR algorithm. In case of CCC, the algorithm trained with 15 components was preferred among others because it provided estimates with comparable validation metrics but lower CV values. In general, it is possible to observe a trend between values of crop trait estimates and their retrieval accuracy: higher estimates are retrieved with higher accuracy (lower CV values). This trend is more significant for nitrogen than chlorophyll, however. The best performing HAL models for LCC, LNC, CCC and CNC were then applied to CHIME-like images acquired on 7 and 30 July 2018, to generate the maps of corresponding crop traits.

**Figure 5:**
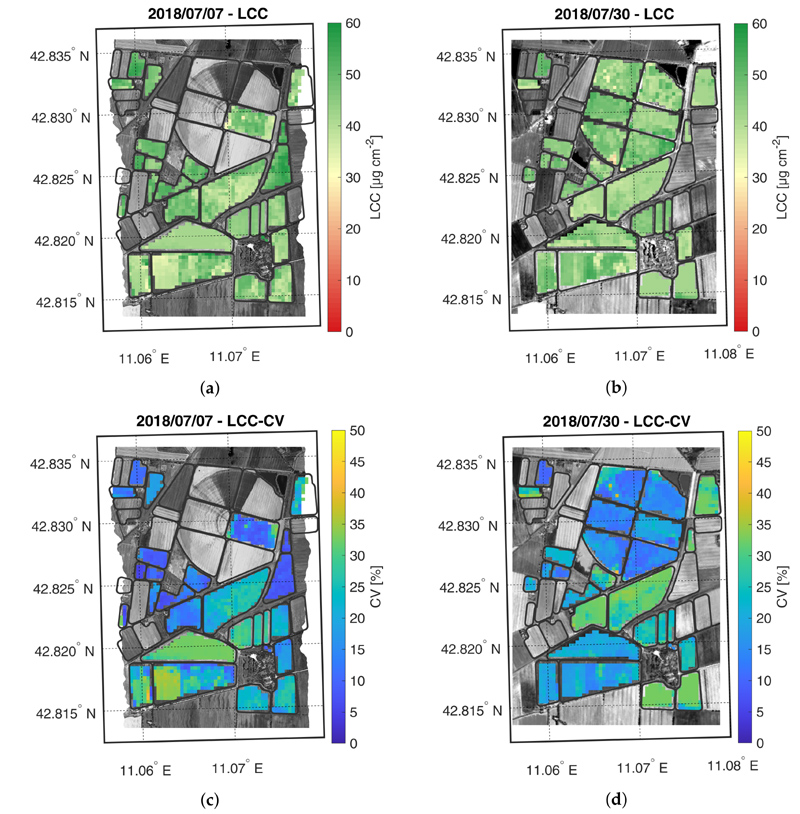
Maps of estimated LCC (a,b) and related CV (c,d) generated from synthetic CHIME-like images, simulated from the Hyplant-DUAL dataset acquired on 7 and 30 July 2018.

**Figure 6:**
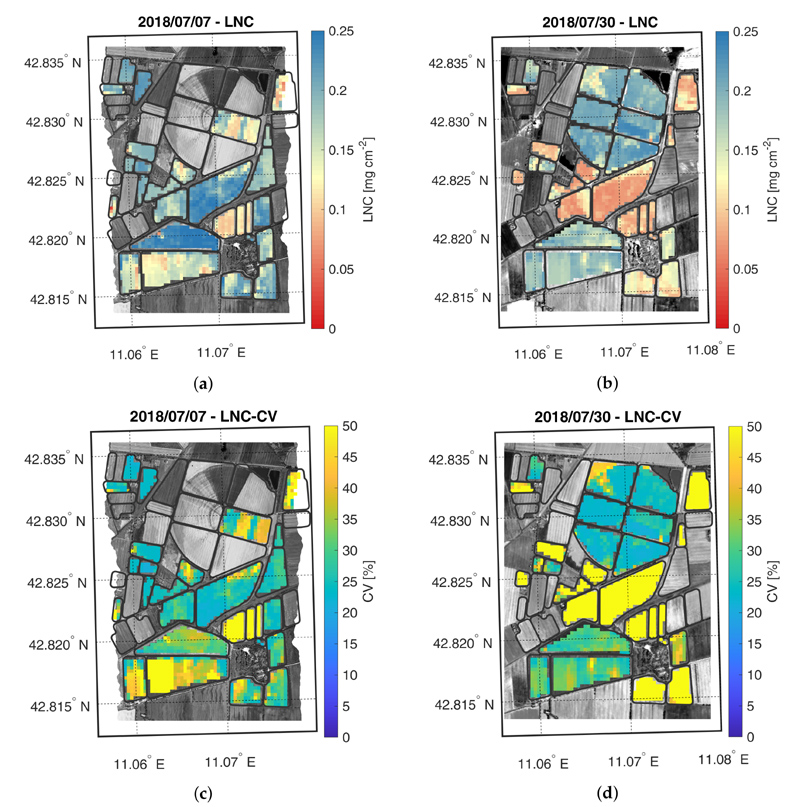
Maps of estimated LNC (a,b) and related CV (c,d) generated from synthetic CHIME-like images, simulated from the Hyplant-DUAL dataset acquired on 7 and 30 July 2018.

**Figure 7:**
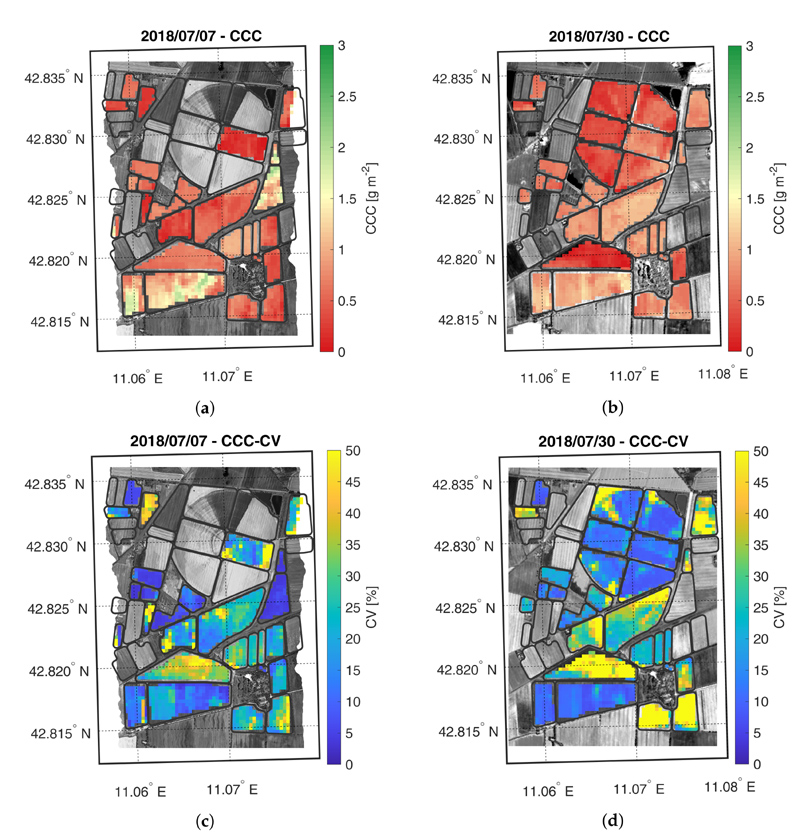
Maps of estimated CCC (a,b) and related CV (c,d) generated from synthetic CHIME-like images, simulated from the Hyplant-DUAL dataset acquired on 7 and 30 July 2018.

**Figure 8:**
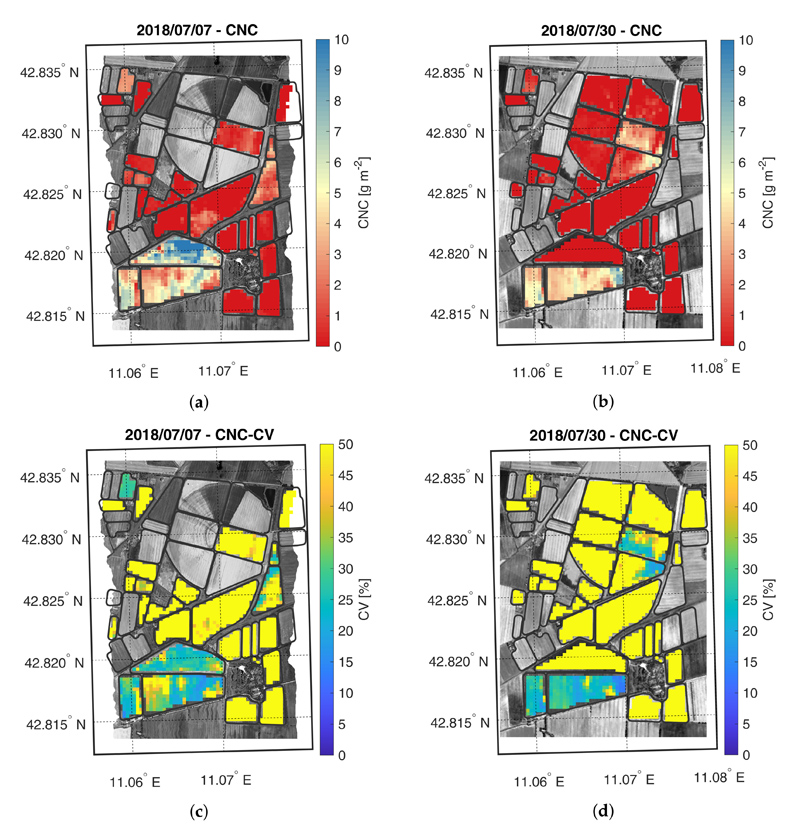
Maps of estimated CNC (a,b) and related CV (c,d) generated from synthetic CHIME-like images, simulated from the Hyplant-DUAL dataset acquired on 7 and 30 July 2018.

The crop trait maps for LCC, LNC, CCC and CNC are reported in Figures 5–8, as well as their corresponding CV maps. The fields with an NDVI value lower than 0.3 were excluded from the maps as they were considered bare soil. All the maps show retrieved values within the range observed during the field campaigns. In general, the trend highlighted in the validation scatter plots is confirmed by the maps, where low trait values are estimated with lower accuracy than higher values.

The LCC maps are reported in Figure 5. Both LFC and HFC fields show an increase in the average chlorophyll content from 7 to 30 July. On 7 July, the HFC field shows retrieved values comparable to the estimates obtained on the only LFC sector with sufficiently grown maize plants (central sector on west side of LFC field). The lower values in the middle part of HFC field were probably underestimated, presenting a lower accuracy estimation (i.e., CV > 30–35%) than the rest of the field. On 30 July, LCC was accurately estimated in both fields (i.e., CV < 20%).

The LNC maps, displayed in Figure 6, show a trend similar to the LCC maps. LNC values increase between the two acquisition dates, for both maize fields. Similarly to LCC, LNC was estimated more accurately during the second date in the two test fields. It is interesting to notice that even though LFC field still presented small maize plants during the second overpass date, the retrievals were generally accurate. However, as shown in the validation scatter plots (Figure 4), LNC was generally estimated less accurately than LCC (i.e., CV ≈ 25–30% and CV < 20% for LNC and LCC, respectively).

The CCC maps are shown in Figure 7. As expected, on the first date, the HFC field shows higher CCC values than the LFC field. On the second date, the CCC maps of the two fields tend to have more similar values. Differently from LCC, the average CCC value in the HFC field decreases between the two acquisitions, suggesting a decrease of the LAI values, in agreement with the phenological phase of maize already in the reproductive growth stage.

The CNC maps, displayed in Figure 8, show a similar trend to their leaf counterpart. On the first acquisition date, the HFC field average value is higher than the LFC average value and these values increase from first to the second acquisition. Compared to CCC, the CNC values were estimated with lower accuracy as shown by CV maps, highlighting possible model flaws, especially for low CNC values.

## 4 Discussion

### 4.1 Crop Trait Retrieval

The results achieved in this study revealed that both hybrid approaches, with and without active learning, are able to estimate chlorophyll and nitrogen with high accuracy at canopy level. Conversely, only HAL was able to accurately retrieve LCC and LNC. The HYB approach provided very poor results, confirming the complexity of trait retrieval at leaf level from canopy reflectance, as highlighted by previous studies [25,64–67]. This can be due to a number of confounding factors, including canopy structure, illumination/viewing geometry and background [25,68–70]. This especially holds true in conditions of sparse canopy cover and/or row crops, where the turbid medium assumptions of 4SAIL are violated, as in the case of LFC field.

At leaf level, the HYB approach using GPR for LCC retrieval provided extremely poor results (R2 = 0.00, RMSE = 13.02–18.39 μg cm^−2^) for all tested dimensionality reductions. Nonetheless, previous studies on LCC showed moderately good results applying the HYB approach to Sentinel-2 using either variational heteroscedastic GPR (R^2^ = 0.47, RMSE = 6.48 μg cm^−2^; [71]) or random forest (R^2^ = 0.26, RMSE = 8.88 μg cm^−2^; [45]). On the other hand, the HAL approach, using CBD as the best AL algorithm, provided accurate estimations (R^2^ = 0.72, RMSE = 3.31 μg cm^−2^). Other studies showed lower performance applying HAL and GPR for the retrieval of LCC in winter wheat with Landsat-8 (RMSE = 12.43 μg cm^−2^ [26]), or multi-crops with PRISMA data (R^2^ = 0.67, RMSE = 5.88 μg cm^−2^ [35]). Moreover, excellent accuracy was obtained applying HAL and kernel ridge regression to synthetic Sentinel-3 data, simulated from PROSAIL (R^2^ = 0.98; [31]).

In case of LNC, HYB with GPR was unable to identify a good model for this trait. One possible reason could be the limited sensitivity of reflectance spectra to leaf protein content, especially in fresh leaves ([24,72]). Nonetheless, HAL using PCA with 10 components and RSAL as AL method provided good results (R^2^ = 0.56, RMSE = 0.02 mg cm^−2^), comparable to other studies using either physically based or HAL approaches. Wang et al. [23] indirectly estimated LNC applying multi-regression models to LCC, leaf mass per area (LMA) and equivalent water content (EWT) predicted from the inversion of PROSPECT-4 model, obtaining good accuracy (R^2^ = 0.58, RMSE = 0.04 mg cm^−2^). An LUT-based inversion approach, exploiting PROSPECT-5 coupled to INFORM model, was used in Wang et al. [25] to estimate LNC (R^2^ = 0.46, RMSE = 0.04 mg cm^−2^) in a forest environment from HySpex airborne sensor. More recently, HAL provided very good LNC estimates (R^2^ = 0.87, RMSE = 0.01 mg cm^−2^ [35]) from PRISMA data, in a multi-crops farming area.

At canopy level, HYB estimations of CCC (R^2^ = 0.79, RMSE = 0.38 g m^−2^) are comparable to previous studies (all based on Sentinel-2) or slightly better in terms of RMSE. Both Upreti et al. [45] and Estévez et al. [71] used PROSAIL as RTM but different ML algorithms: PLSR (R^2^ = 0.74, RMSE = 0.40 g m^−2^) and VHGPR (R^2^ = 0.85, RMSE = 0.39 g m^−2^), respectively. Brown et al. [73] coupled PROSAIL and INFORM to train an artificial neural network model optimized for forestry environments achieving R^2^ = 0.69 and RMSE = 0.52 g m^−2^. HAL provided better CCC estimates (R^2^ = 0.88, RMSE = 0.21 g m^−2^) than HYB and also better than Tagliabue et al. [35] (R^2^ = 0.82, RMSE = 0.36 g m^−2^).

Considering CNC, HYB results obtained in this study (R^2^ = 0.84, RMSE = 1.10 g m^−2^) are comparable to those achieved in Berger et al. [74] using VHGPR for the estimation of aboveground nitrogen content in winter wheat and maize (R^2^ = 0.86, RMSE = 2.15 g m^−2^). It is worth noting that in Berger et al. [74], the CNC was better correlated with leaves plus stalks than only to leaves as in this work. Similarly to other traits, HAL improved the retrieval accuracy (with respect to HYB) for CNC (R^2^ = 0.93, RMSE = 0.71 g m^−2^), achieving slightly superior results than previous studies. In Verrelst et al. [32], a training database generated from PROSAIL-PRO was optimized through AL and the reduced LUT was used to train the VHGPR model for CNC estimation in winter wheat, maize and grass (R^2^ = 0.92, RMSE = 1.84 g m^−2^). Finally, both Verrelst et al. [34] and Tagliabue et al. [35] applied a similar HAL approach to actual PRISMA data for CNC estimations in winter wheat and maize (R^2^ = 0.72, RMSE = 3.26 g m^−2^) and multi-crops (R^2^ = 0.92, RMSE = 0.96 g m^−2^), respectively.

### 4.2 Impact of Dimensionality Reduction

Regarding the dimensionality reduction, the results reported in Table 4 and Figure 3 show different trends at leaf and canopy level. In particular, HYB approach provided accurate CCC estimations for all tested DR configurations (R^2^ = 0.79–0.81, RMSE = 0.38–0.54 g m^−2^). CNC showed a similar trend, achieving good results for all PCA components (R^2^ = 0.83–0.84, RMSE = 1.10–1.31 g m^−2^). Regarding HAL approach, very good results were achieved at canopy level, for both CCC and CNC, considering all the tested combinations of spectral (PCA components) and sample (AL algorithms) reductions (R^2^ = 0.86–0.87, RMSE = 0.21–0.22 g m^−2^ and R^2^ = 0.92–0.93, RMSE = 0.72–0.76 g m^−2^ for CCC and CNC, respectively). Conversely, only some DR configurations provided good results at leaf level, as shown in Figure 3: CBD and EBD with PCA10, PCA20 and noPCA for LCC; PAL with PCA15 and RSAL with both PCA10 and PCA15 for LNC.

In a recent survey about the use of HAL approach for the estimation of terrestrial vegetation traits from EO data, Berger et al. [33] identified six peer-reviewed articles fulfilling the search requirements: Verrelst et al. [31], Upreti et al. [45], Verrelst et al. [32], Upreti et al. [75], Zhou et al. [26] and Pipia et al. [76]. In addition to the abovementioned articles, two other papers were published later, on the same topic: Verrelst et al. [34] and Tagliabue et al. [35]. These studies showed that no AL method outperforms the others for a single crop trait: different best-performing ALs are obtained in different contexts, even though these differences are often small. Considering LCC, for example, all AL methods tested by Verrelst et al. [31] provided R^2^ > 0.94, whereas EBD achieved only slightly higher performance than ABD and CBD for the case study presented in Upreti et al. [45]. In Zhou et al. [26] the combination of entropy query by bagging AL and GPR showed the best accuracy for LCC estimation (RMSE = 12.43 μg cm^−2^, rRMSE = 21.77%), whereas ABD reached the best performance (R^2^ > 0.67, RMSE = 5.88 μg cm^−2^, nRMSE = 11.70%) in Tagliabue et al. [35]. Considering the above results, both spectral and sampling dimensionality reductions are critical factors for the crop trait retrieval process. Although this does not represent an issue at canopy level, where all the tested combinations provided similar results, it seems to be relevant when working at leaf level. Therefore, a priori choice of the number of PCA components and/or AL heuristics might lead to sub-optimal results, especially at leaf level.

Beside PCA, different dimensionality reduction approaches have been proposed in the literature within the hybrid framework. Examples of such approaches include band selection either by expert-based/physically based knowledge [25,74] or automatic band selection tools [74,77,78]. With respect to PCA, these approaches have the advantage of an easier interpretation of the band physiological meaning, even though this is at the expense of the information content available over the full spectral range. In this regard, it would be worth exploiting a comprehensive dataset (multi-season, multi-site and multi-crop) for a systematic comparison of the performance of different DR approaches, including automatic band selection tool, PCA and physically based approaches, to evaluate the best strategy and its transferability in different contexts.

### 4.3 Operational Use of the Hybrid Approach in the Framework of CHIME Mission

The presented work was performed within the ESA CHIME Mission Requirements Consolidation (MRC) study. This study was devoted to evaluate retrieval methods to assess several priority variables, identified by the CHIME mission advisory group among the thematic fields of vegetation, soil and raw materials. The findings from CHIME-MRC will support the development of the CHIME end-to-end (E2E) mission performance simulator. The simulator, whose development already started within the ESA CHIME-E2E study, is able to accurately simulate all the steps required in the EO data processing chain, starting from data acquisition up to final surface parameter maps, which also includes the crop traits presented here [79]. In the next years, during the CHIME mission preparation phase, the simulator will be further expanded and improved upon until the launch of the satellite, within the CHIME-E2E mission performance simulator project [34]. In particular, one of the main features of the simulator is the possibility to validate the generated products against reference input data, allowing its validation and tuning, exploiting new data from planned campaigns [79]. This feature is extremely important considering that, differently from HYB, which is a method independent from external data, HAL relies on in situ measurements for the sampling optimization step. While this improves the retrieval performance, it might limit the model transferability if the reference dataset is not representative of broad crop conditions. In this regard, the uncertainty map associated with crop trait estimates is an interesting GPR feature. Uncertainty maps, representing a measure of the model retrieval performance, can be used to assess map quality and to mask less reliable trait estimates (e.g., CV > 20%). Moreover, this GPR feature can provide useful information about crop types or conditions not taken into account during the AL phase as well as other non-vegetated surfaces which should be included in the training dataset for global mapping. The latter is especially important when the hybrid approach is applied within an operational framework. In such a case, the addition of non-vegetated spectra to the training dataset will increase the reliability of the estimates when mapping a whole EO image, which usually includes any kind of surface [34]. To date, only Tagliabue et al. [35] performed a rigorous validation of HAL performance: the authors trained a HAL model with a dataset acquired in 2020 and used an independent dataset acquired in the same study area in 2021 for the validation. The retrieval accuracy for the two years showed almost comparable performance for chlorophyll at both canopy (CCC: R^2^ = 0.82, nRMSE = 10.2% in 2020 and R^2^ = 0.84, nRMSE = 18.5% in 2021) and leaf level (LCC: R^2^ = 0.67, nRMSE = 11.7% in 2020 and R^2^ = 0.62, nRMSE = 27.9% in 2021), but worse performance for nitrogen (CNC: R^2^ = 0.92, nRMSE = 5.5% in 2020 and R^2^ = 0.79, nRMSE = 23.7% in 2021), especially at leaf level (LNC: R^2^ = 0.87, nRMSE = 7.46% in 2020 and R^2^ = 0.35, nRMSE = 28.4% in 2021), requiring additional improvements. The high accuracy and consistency of the results obtained for most traits supports the use of the HAL approach applied to spaceborne imaging spectroscopy for crop monitoring. Future studies should be devoted to test HAL performance in diverse locations and/or different growing seasons, broadening vegetation types and conditions, to improve its generalization for an operational use within the CHIME mission.

## 5 Conclusions

This work evaluated the potential of the hybrid approach for the retrieval of maize traits (chlorophyll and nitrogen at leaf and canopy level) within the framework of the future space-borne hyperspectral mission CHIME. The standard hybrid approach HYB and its recent variant HAL, used to optimize the LUT through different active learning heuristics, were tested in combination with several feature dimensionality reductions based on PCA.

The analysis of retrieval accuracy proved that both hybrid approaches are able to accurately estimate chlorophyll and nitrogen content at canopy level. Conversely, only HAL was able to accurately retrieve the crop traits at leaf level. Regarding the dimensionality reduction, the results showed that an a priori choice of the number of PCA components and/or AL heuristics is a critical factor in the trait retrieval process and might lead to sub-optimal results, especially at leaf level.

In conclusion, the results obtained in this and previous studies proved that crops traits can be successfully assessed from spaceborne imaging spectroscopy. However, it is worth noting that, whereas HYB is an approach fully independent from external data, HAL relies on measured data to perform the active learning step. In either case, the retrieval strategies need to be further investigated across different years, sites and crop types in order to improve the actual transferability to other contexts. This will help to understand the real potential of this approach, within the operational framework of the future CHIME mission.

## Figures and Tables

**Table 1:** List of crop traits used in this study, units, number of available samples, average, standard deviation and the method used to derive them.

Trait	Unit	Samples	Mean	St.Dev.	Method
LCC	μg cm^−2^	87	43.31	6.29	SPAD-LCC regression
LNC	mg cm^−2^	31	0.15	0.03	Lab. measurements
CCC	g m^−2^	87	0.75	0.61	Derived from LCC and LAI
CNC	g m^−2^	31	3.87	2.82	Derived from LNC and LAI

**Table 2:** Technical features of HyPlant-DUAL acquisitions over the study area, during the 2018 ESA FLEXSENSE campaign held in Grosseto.

Date	Lines	Tot. Length	Tot. Area	Swath	GSD
7 July 2018	6	∼7 km	∼18 km^2^	400 m	1 m
30 July 2018	4	∼8 km	∼20 km^2^	1800 m	4.5 m

**Table 3:** List of PROSAIL-PRO input variables used to generate the LUT. Inputs variables were randomly sampled according to the reported distributions and ranges.

	Param.	Description	Unit	PDF	Range ^1^	
PROSPECT-PRO	N	Structural parameter	-	Normal	1.4	0.14
	Cab	Chlorophyll content ^2^	μg cm^−2^	Normal	41.5	8.8
	Ccx	Carotenoid content ^2^	μg cm^−2^	Normal	7.32	1.5
	Cantd	Antdocyanin content	μg cm^−2^	Normal	0.0	0.0
	Cbp	Brown pigment content	μg cm^−2^	Normal	0.0	0.0
	Cw	Water content ^2^	mg cm^−2^	Normal	12.92	1.91
	Cp	Protein content ^2^	g cm^−2^	Uniform	0.0	0.001
	CBC	Carbon-Based Constituents	g cm^−2^	Uniform	0.003	0.006
4SAIL	ALA	Average Leaf Angle ^2^	°	Normal	49.0	4.9
	LAI	Leaf Area Index ^2^	m^2^ m^−2^	Normal	1.77	1.4
	HOT	Hot spot parameter	m m^−1^	Normal	0.01	0.001
	SZA	Solar Zenith Angle ^2^	°	Uniform	26	30
	OZA	Observer Zenith Angle	°	Uniform	0	0
	RAA	Relative Azimuth Angle	°	Uniform	0	0
	BG	Soil Spectra ^2^	-	Uniform	2	4

1 min and max values in case of Uniform PDF; *μ* and *σ* values in case of Normal PDF.

2 Ranges set according to values measured in this study.

**Table 4:** Goodness-of-fit metrics for crop trait retrieval using standard hybrid approach (HYB) or hybrid with AL approach (HAL). Statistics are expressed as coefficient of determination (R^2^), Root Mean Square Error (RMSE) and normalized RMSE (nRMSE).

		HYB			HAL			
	DR	R^2^	RMSE	nRMSE	R^2^	RMSE	nRMSE	AL
LCC	PCA05	0.00	18.39	62.99%	0.27	5.58	19.11%	CBD
	PCA10	0.00	16.91	57.91%	0.69	3.51	12.00%	CBD
	PCA15	0.00	16.98	58.16%	0.41	4.80	16.44%	EBD
	PCA20	0.00	15.98	54.74%	0.72	3.31	11.32%	CBD
LNC	PCA05	0.00	0.07	54.43%	0.02	0.03	24.28%	EBD
	PCA10	0.06	0.38	302.7%	0.56	0.02	16.36%	RSAL
	PCA15	0.24	0.18	125.9%	0.55	0.02	16.69%	RSAL
	PCA20	0.00	0.16	125.6%	0.06	0.03	24.11%	PAL
CCC	PCA05	0.79	0.38	13.40%	0.87	0.22	7.68%	ABD
	PCA10	0.79	0.50	17.70%	0.88	0.21	7.54%	CBD
	PCA15	0.80	0.54	18.99%	0.87	0.22	7.89%	PAL
	PCA20	0.81	0.54	19.01%	0.86	0.23	8.05%	CBD
CNC	PCA05	0.83	1.31	14.21%	0.92	0.77	8.35%	EBD
	PCA10	0.84	1.10	11.93%	0.93	0.71	7.69%	CBD
	PCA15	0.83	1.17	12.66%	0.93	0.72	7.77%	EBD
	PCA20	0.84	1.12	12.18%	0.93	0.74	8.06%	RSAL

## Data Availability

The data presented in this study are available on request from the corresponding author.
